# Deciphering Multiple Sclerosis Progression

**DOI:** 10.3389/fneur.2021.608491

**Published:** 2021-04-07

**Authors:** Virginia Meca-Lallana, Leticia Berenguer-Ruiz, Joan Carreres-Polo, Sara Eichau-Madueño, Jaime Ferrer-Lozano, Lucía Forero, Yolanda Higueras, Nieves Téllez Lara, Angela Vidal-Jordana, Francisco Carlos Pérez-Miralles

**Affiliations:** ^1^Multiple Sclerosis Unit, Neurology Department, Fundación de Investigación Biomédica, Hospital Universitario de la Princesa, Madrid, Spain; ^2^Neurology Department, Hospital Marina Baixa, Alicante, Spain; ^3^Neuroradiology Section, Radiology Department, Hospital Universitari i Politècnic La Fe, Valencia, Spain; ^4^Multiple Sclerosis CSUR Unit, Neurology Department, Hospital Universitario Virgen Macarena, Seville, Spain; ^5^Department of Pathology, Hospital Universitari i Politècnic La Fe, Valencia, Spain; ^6^Neurology Department, Hospital Puerta del Mar, Cádiz, Spain; ^7^Neurology Department, Instituto de Investigación Sanitaria Gregorio Marañón (IISGM), Hospital Universitario Gregorio Marañón, Madrid, Spain; ^8^Department of Experimental Psychology, Cognitive Processes and Speech Therapy, Universidad Complutense, Madrid, Spain; ^9^Neurology Department, Hospital Clínico Universitario de Valladolid, Valladolid, Spain; ^10^Neurology/Neuroimmunology Department, Centre d'Esclerosi Múltiple de Catalunya (Cemcat), Hospital Universitari Vall d'Hebron, Barcelona, Spain; ^11^Neuroimmunology Unit, Neurology Department, Hospital Universitari i Politècnic La Fe, Valencia, Spain; ^12^Department of Medicine, University of València, Valencia, Spain

**Keywords:** multiple sclerosis, neurodegeneration, progressive multiple sclerosis, neurofilament, MRI

## Abstract

Multiple sclerosis (MS) is primarily an inflammatory and degenerative disease of the central nervous system, triggered by unknown environmental factors in patients with predisposing genetic risk profiles. The prevention of neurological disability is one of the essential goals to be achieved in a patient with MS. However, the pathogenic mechanisms driving the progressive phase of the disease remain unknown. It was described that the pathophysiological mechanisms associated with disease progression are present from disease onset. In daily practice, there is a lack of clinical, radiological, or biological markers that favor an early detection of the disease's progression. Different definitions of disability progression were used in clinical trials. According to the most descriptive, progression was defined as a minimum increase in the Expanded Disability Status Scale (EDSS) of 1.5, 1.0, or 0.5 from a baseline level of 0, 1.0–5.0, and 5.5, respectively. Nevertheless, the EDSS is not the most sensitive scale to assess progression, and there is no consensus regarding any specific diagnostic criteria for disability progression. This review document discusses the current pathophysiological concepts associated with MS progression, the different measurement strategies, the biomarkers associated with disability progression, and the available pharmacologic therapeutic approaches.

## Introduction

Multiple sclerosis (MS) has been classically defined as a demyelinating disease of the central nervous system (CNS), with preferential involvement of the white matter. In MS two pathological phenomena converge. There is an inflammatory event, responsible for clinical relapses and demyelination plaques in the CNS; and there is a neurodegenerative phenomenon responsible for progressive disability worsening.

Most patients with MS have symptoms of acute/subacute focal neurological deficit, which have come to be called “relapses” or “attacks,” with repetition of episodes in various locations of the CNS over time. This more frequent form of MS course is called “relapsing-remitting” (RRMS). After several years, 35–50% of patients initially classified as RRMS go into a phase characterized by slowly progressive neurological deterioration independent of previous inflammatory activity (1–4), which is commonly referred to as the “secondary progressive” phase (SPMS). Approximately 15% of patients with MS begin a slowly progressive deterioration from the start in the absence of detectable clinical relapses, a course that has been called “primary progressive” (PPMS). A fourth category, called “recurrent progressive,” described those who, although initially behaving the same as patients with PPMS, develop some relapses during the course (5). This classification was revised and modified, so that the “recurrent progressive” category disappears, and activity criteria are defined, measured either by the presence of clinical relapses or activity on MRI, or by progression of disability (6).

The transition to a progressive course of the disease seems to be age-dependent. Although recent natural history studies show a lower percentage of patients transitioning from RRMS to SPMS, the age of transition is surprisingly immutable, around the forties (4, 7). With aging, comorbidities also play a role, and are intimately related to disability and health care resources consumption (8–10).

The neurodegenerative process, historically, was not included in the clinical diagnostic criteria of MS, with the exception of the 1965 Schumacher criteria, where two patterns of clinical involvement of MS were recognized: one with episodes of clinical deterioration separated by a month or more from each other, with a duration of at least 24 h (similar to the current definition of relapses), and a second pattern with a gradual clinical worsening over a period of 6 months or longer (11). In 1983, the Poser diagnosis criteria excluded progressive forms of MS, in part due to lack of reliable radiological or biological markers ruling out alternative diagnoses (i.e., progressive myelopathy or spinocerebellar syndrome) at that time (12). Although the currently recognized clinical forms of progressive MS are only defined based on clinical criteria (6), the emergence of magnetic resonance imaging (MRI) techniques allowed a better characterization of progressive MS and its differentiation between PPMS and SPMS (13). Since 2001, PPMS has been included explicitly in the McDonald criteria and its updates (14–17). Nowadays, both PPMS and SPMS are usually referred to as progressive MS (PMS).

Prevention of irreversible disability is one of the essential clinical goals in a patient with MS. However, in daily practice, there is still a lack of clinical, radiological, or biological markers that favor an early detection of the progressive course of the disease, and also there is no expert consensus on specific diagnostic criteria for disability progression (18).

## Objectives

The Progression Working Group is part of the EMDAT study group (Esclerosis Múltiple Disease Activity Task Force). This is a multidisciplinary team consisting of eight neurologists (AVJ, FCPM, LBR, LFD, NTL, SEM, VML), a neuropsychologist (YH), an anatomopathologist (JFL) and a neuroradiologist (JCP) from different Health Centers in Spain, all experts in the management of patients with MS. This group aimed to elaborate a review document based on the current concepts of MS progression.

## Materials and Methods

**Figure 1:**
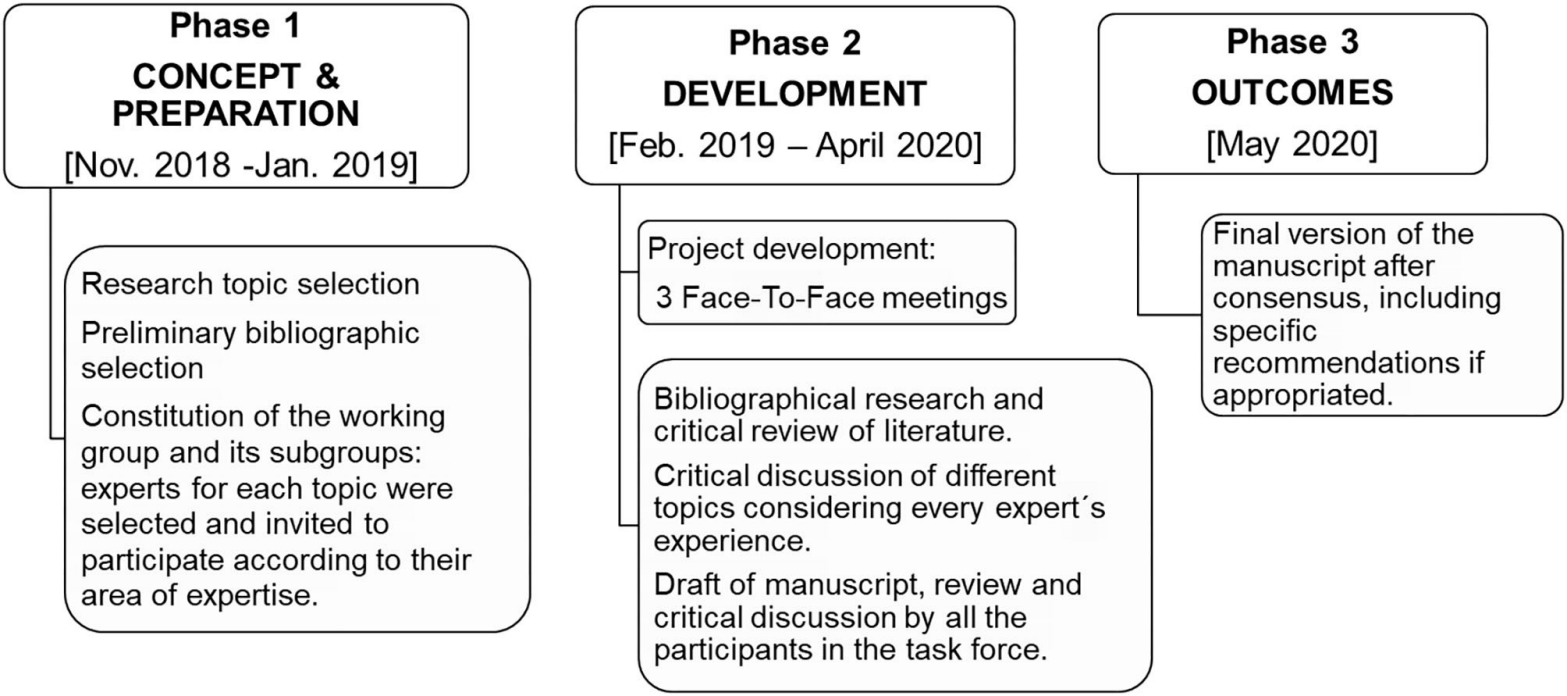
Overall workflow, timing, and expert group/subgroups composition.

The EMDAT Progression Working Group was divided into two subgroups with the aim to address different topics in MS progression. The first group focused on clinical and cognitive characteristics (VML, LBR, LFD, NTL, YH); the second group focused on paraclinical items: pathology, pathophysiology, and imaging markers (FCPM, AVJ, JCP, JFL, SEM). A non-systematic review document was elaborated after discussion among the participants, based on the review of the literature and the experience of each expert. The workflow for this document is detailed in Figure 1.

## Pathology and Pathophysiology of Progressive MS

The combination of primary perivenular demyelination, loss of oligodendrocytes, inflammation, and remyelinated spots, distinguishes MS from other demyelinating disorders of the CNS (19). These features, as well as neurodegenerative changes resulting from neuronal damage, are present in all the evolutional stages of MS (20), and are always associated with B and T-cell mediated immunity, macrophages, and microglia. Lesions in MS can affect both white and gray matter of the brain and spinal cord.

The histopathological differences between relapsing and progressive forms of MS are mainly quantitative (19). Thus, relapsing MS is characterized by the predominance of confluent plaques of active demyelination, located mainly in the white matter and in areas of high venous density. They present a central inflammatory region with abundant T lymphocytes (especially CD8^+^), macrophages and are associated with an accumulation of microglia. In contrast, PMS shows a higher number of slowly expanding lesions distributed in areas with less blood perfusion. Histologically these lesions present an inflammatory margin of macrophages and activated microglia that surrounds a demyelinated and inactive center. Damage to the blood-brain barrier, which is very characteristic in relapsing-remitting MS (RRMS), is less evident in PMS, leading to partial compartmentalization of inflammation.

However, in other study qualitative differences and heterogeneity have been observed in MS lesions between different patients, defining a series of patterns of MS lesions (21). Normally, in the same subject, all active plaques correspond to the same pattern, although these patterns may be different between subjects (21, 22). However, other studies have not confirmed this fact, suggesting that, rather than interindividual, heterogeneity is dependent on time and the stage of evolution of the lesions in the same individual (23, 24). Although these differences did not clearly characterize the type of MS, it may be important to also take it into consideration.

Lesions in the gray matter predominate in PMS, especially areas of subpial cortical demyelination (25). These lesions also present inflammation, but to a lesser degree compared to white matter plaques. They can be associated with different pathological features, like ectopic lymphoid follicles, neuronal and glial cell loss, and extensive remyelination zones (26). Finally, in PMS, there is a diffuse inflammatory infiltration in the “normal-appearing” white matter, where activation of microglia, axonal damage, and reactive gliosis are seen (25).

The mechanisms that link alterations of the immune response and cerebral demyelination remain unknown. Demyelination might be associated with local microenvironmental factors or soluble factors produced by meningeal infiltrates. Demyelination could be triggered by a specific antibody, although the particular antigen targeting the myelin sheath has yet to be identified. On the other hand, genetic studies reveal activation in the oxidation of neurons, myelin, and oligodendrocytes, associated with mitochondrial damage (27, 28). The mitochondrial alteration would imply an energy deficit in the form of “virtual hypoxia” (29), that would amplify oxidative damage causing an imbalance in cell ion exchange through mechanisms linked to calcium channels. Finally, extracellular iron, released by injured oligodendrocytes and microglia, increases tissue susceptibility to oxidative damage caused by free radicals. All these processes may play an important role in the progressive forms of MS when cellular aging and the number of accumulated lesions exceed the brain adaptive mechanisms (30).

## Measurement Strategies in MS Progression

### Defining Progression in MS

In most MS clinical trials, the definition of progression is based on the increase in the Expanded Disability Status Scale (EDSS) (31) confirmed over time (18). The current concept of MS progression recommended by the European Medicines Agency (32) (Table 1), and the most widely used in clinical trials, comes from a meta-analysis of four clinical trials. Based on the patient's baseline EDSS, it establishes two different strata when defining progression. If the patient's baseline EDSS is below 6, only ≥1 point of sustained impairment is needed for progression to be considered, whereas ≥0.5 points are required if baseline EDSS is higher than 6.0 (34). Later, an additional stratification of the scale was suggested, so in patients with EDSS = 0, an increase of ≥1.5 points would be needed (18), together with a major affection on the pyramidal functional system of ≥2 (33).

**Table 1:** Definitions used to diagnose disease progression in MS.

	**Definition of progression (EDSS worsening from baseline)**	**Time to confirm disease progression**	**Population studied**
European Medicines Agency (32)	1.0 point if EDSS ≤ 5.5 0.5 point if EDSS ≥ 6.0	6 months	MS
Kalincik et al. (18)	1.0 point if EDSS ≤ 5.5 0.5 point if EDSS ≥ 6,0	12 or 24 months	PMS (PPMS and SPMS)
Lorscheider et al. (33)	1.0 point if EDSS ≤ 5.5 0.5 point if EDSS ≥ 6.0 FSS ≥ 2	3 months if basal EDSS ≥ 4.0	SPMS

*EDSS, Expanded Disability Status Scale; FSS, Functional Systems Score; PMS, progressive multiple sclerosis; PPMS, primary progressive multiple sclerosis; SPMS, secondary progressive multiple sclerosis*.

On the other hand, there is no specific period of time for the identification of a progressive course of the MS or confirmation of its diagnosis (Table 1). This is an essential issue in the relapsing form of the disease, since the time when a patient converts to SPMS is unclear. Furthermore, during the natural course of the disease, patients in the progressive phase may continue to have relapses combined with periods of stabilization or slight clinical improvement, making thus the diagnosis more difficult (6).

It was suggested that the “transition” from the relapsing phase of the disease to the progressive one is only an artifact caused by the low sensitivity to detect minor clinical changes by the current clinical scales (35) and the brain plasticity that can compensate for clinical deficits at disease onset (36). Nevertheless, it has been proposed that the pathophysiological mechanisms associated with disease progression are probably present from the disease onset in RRMS patients, but they are often silent (37–39).

To confirm disability progression, a patient's clinical worsening should be reassessed after a variable period of time (18, 40). In relapsing MS, the irreversible progression of disability can be biased because of the transient deterioration associated with relapses. For this reason, it was proposed to use different clinical outcome measures to assess progression and relapses (41). The clinical improvement following a relapse usually occurs after 2 months, although recovery can occur longer than 12 months in some patients (42). For this reason, to improve sensitivity when assessing disability progression, it is suggested to evaluate patients after 1 month of clinical stability following a relapse, avoiding thus possible bias (43).

A confirmed increase in the EDSS measured at 3 or 6 months may not provide an accurate estimate of long-term disease outcomes. In this context, it was described that up to 30% of patients with a confirmed progression at 3–6 months follow up might have clinical improvements if they are reevaluated after 12–24 months, especially younger patients or if small changes in the EDSS took place (18). On the other hand, although a longer time for the disability confirmation (i.e., 12–24 months) would increase the irreversibility sensitivity, this could delay therapeutic interventions.

Recently, a novel definition of progressive MS has been proposed. It shortened the time to diagnosis in routine clinical practice (Table 1) and was introduced after comparing 576 different definitions of EMSP from a vast European registry. According to this new definition, if the patient presents with an EDSS ≥ 4 and has a functional impairment of the pyramidal system of ≥2, the clinical reassessment to confirm disability can be done during the 3 months follow up (33). Therefore, in patients with an EDSS <4.0, the diagnosis of irreversible disability progression should be made with caution, and a longer follow-up is suggested to safely confirm it (i.e., 6, 12, or 24 months) (18, 40).

### Physician-Oriented Measures in Progressive MS

To date, no particular scale is considered as the gold standard to assess clinical progression in MS. Due to the high variability of MS, it seems unlikely that a single evaluation strategy will be adequate to meet all evaluation objectives in different patients. Because of this, it is necessary to use multidimensional outcome measures. Different scales would probably be required at different stages of the disease over time (41).

The EDSS is the most widely used scale in MS. Its main advantage is its familiarity, widespread use, and the information gathered on its validity and reliability (41). Although this scale is based on neurological examination and is clinically relevant, it has some disadvantages: (a) it presents an intra- and inter-evaluator variability that can reach up to 40% due to its low precision in distinguishing amongst mild, moderate and severe symptoms, (b) it has subjective items, such as bowel and bladder dysfunction, (c) the scale is asymmetric, assigning a relatively higher weight to patient's locomotion, and (d) the times spent in each level of the scale is variable, being higher in the upper strata (18, 41).

The stratification of the EDSS has been used to improve its precision in detecting progression. The presence of two strata (an increase of one point for EDSS between 0 and 5.5, or 0.5 points above EDSS of 5.5) could overestimate the progression compared to the presence of three strata (EDSS increase of 1.5 points if the baseline EDSS is 0, an increase of one point if the baseline is between 1 and 5.5, or increase of 0.5 points if the baseline is above 5.5). However, it must be noted that the EDSS has more stability at the highest levels of the scale and, therefore, the clinical worsening needed to consider progression would be higher. Consequently, it may be necessary to include additional assessments in patients with higher disability status to improve the progression detection sensitivity (18) since it would not measure small changes at this point (40).

The Neurostatus developed by Kappos, used in the European trial on beta-interferon-1b in the SPMS, includes a neurological examination similar to the EDSS, with extra items added to assess ambulation and vision, to improve sensitivity (41, 44). Nevertheless, in this evaluation, the locomotion also has a significant weight compared to the rest of the functional systems.

An international working group developed the Multiple Sclerosis Functional Composite (MSFC). It is comprised of three performance tests: 9-Hole Peg Test (9HPT) to assess upper limb function, Timed 25-Foot Walk (T25FW) to determinate ambulation, and Paced Auditory Serial Addition Test (PASAT) to assess cognition (45). Since the variables evaluated in these three tests are different, a Z score was selected as a standard metric. The MSFC is objective, multidimensional, standardized, and more sensitive in detecting clinical changes than traditional ordinal scales (41). The disadvantage of this method is related to the use of the Z-score, which orders the patient in the number of standard deviations with respect to a reference population, but does not directly translate the patient's disability status, nor is it easy to interpret changes that may occur in this score (46). However, it has been proven that a worsening of 0.5 in the total score, or >20% in each component, is a clinically significant marker of disease progression (47, 48).

Composite measurements such as “EDSS-plus” have been proposed in response to the multidimensional needs of the disease (49). The aim was to improve the detection of disability progression by adding T25FW and 9HPT to the EDSS. A worsening ≥20% for T25FW and 9HPT, confirmed at 24 weeks, differentiates more accurately between patients who progress to PMS from those who do not. In this respect, rates of disability progression at 24 weeks measured by EDSS-Plus were 59.5%, compared to only 24.7% when standard EDSS was used, suggesting that EDSS-Plus is approximately twice more sensitive than EDSS (49). On the other hand, the ORATORIO trial included the No Evidence of Progression (NEP) endpoint, which was defined as no progression in EDSS, no worsening of ≥20% on 9HPT, or ≥20% on T25FW for 12 weeks (50). In this trial, more than half of the patients who did not show a worsening of disability measured by EDSS experienced a confirmed worsening of ambulation when measured by T25FW. Also, a considerable proportion of patients who did not have a confirmed progression in EDSS, displayed a worse upper limb function when they were evaluated by using the 9HPT (50).

In conclusion, a significant change of ≥20% in the T25W (51), ≥20% 9HPT (52), or ≥7-point change in the low-contrast letter acuity chart (53) should be considered progression of MS.

Finally, the use of new technologies for real-time measurement of patient disability, for example, wrist accelerometers for remote gait measurement are useful despite their limitations and will undoubtedly be applied in future clinical practice to help detect progression (54, 55).

### Patient Self-Administered Scales

The patient-reported outcome (PRO) measures are questionnaires designed for patients to indicate their perceptions regarding their health status, quality of life, and well-being (56). Although PRO measures are not widely used in clinical practice, it has been shown that worse scores are obtained from patients with progressive forms of MS, even in the early stages of the disease (57).

The systematic use of PRO questionnaires could provide additional information on standard disability measurements for a particular patient. Considering the widespread use of digital tools, the use of different PRO tests would not necessarily increase the time spent in the neurologist office. We believe that they can be especially useful for those patients with mild disabilities or for those who complain of clinical deterioration, when that impairment cannot be confirmed with standard physician-oriented measures.

It was described that the PRO test could be used for the assessment of the MS population, based on different randomized controlled trials and observational studies (58) Taking into account different psychometric parameters, such as time of administration and neuropsychological domains to be assessed, as well as the level of the evidence supporting them, our group recommended the use of generic scales that focus on the quality of life such as the Short Form 36 (SF-36) (59) and the European Quality of Life (EQ-5D) scale (60), or specific MS scales as Multiple Sclerosis Quality of Life-54 (MSQol-54) (61). To measure the impact of the disease on the patient's motor skills and cognition, this group recommended the Multiple Sclerosis Impact Scale-29 (MSIS-29) (62). In the case where subjective gait complaints are suspected, the 12 items Multiple Sclerosis Walking Scale (MSWS12) can be used (63). Finally, to objectively assess a patient's fatigue, the Modified Fatigue Impact Scale (MFIS) can be useful (64).

There are also limitations for the PRO questionnaires since there is a lack of validation studies to support their use and no standardized cut-off points in most tests. Also, PRO questionnaires cannot be used for therapeutic decision-making since they do not allow for the confirmation of the progression of the disease.

### Neuropsychological Assessment

Currently, international consensus indicates that clinical follow-up of the MS population should include a neuropsychological status assessment (65, 66). Amato et al. considered that the neuropsychological evaluation is a tool that will help to redefine the clinical activity in MS. Furthermore, it is of fundamental importance when a progression of the disease is suspected (67).

In PMS, there is a higher percentage of patients with cognitive impairment than relapsing forms of the disease (86% in the secondary progressive and 74% in the primary progressive forms). Moreover, the severity of cognitive impairment is higher in PMS, with more cognitive domains affected by the disease (68). Compared to RRMS, in PPMS the cognitive symptoms are more prominent during disease onset, and patients have a worse cognitive decline after 6 years of follow-up (69).

Neuropsychological assessment has three aims in the clinical monitoring of the MS. First, it serves as a measurement of progression. An international consensus recommended an early baseline assessment of cognitive status of newly diagnosed patients and a follow-up reevaluation after 1 or 2 years, depending on the patient's cognitive impairment (65, 68, 70). This assessment can be carried out by using a screening test, such as the Symbol Digit Modality Test (SDMT) (71). However, the use of a more comprehensive evaluation could provide more information regarding other neuropsychological domains, such as cognitive, emotional, and behavioral processes (65, 66). The second aim is to predict cognitive impairment. Studies showed that patients with untimely cognitive impairment are more likely to develop earlier the secondary progressive form of the disease (72). Also, in newly diagnosed patients, alterations in memory and speed of information processing predict physical disability as measured by EDSS at 5 and 7 years (73). Mood disorders during disease onset (i.e., depression or anxiety) also serve as predictors of cognitive changes after 1 year of follow-up (74). Physical fatigue in early phases is related with a rise in disability degree 3 years later. Accordingly, an increase of 10 points in the Fatigue Impact Scale would indicate clinical deterioration (75). The third aim is to certify the progression of the disease. It has been established that a reduction of ≥4 points or a 10% worsening in the Symbol Digit Modality Test without concomitant depression or fatigue, can be considered clinical deterioration (76). However, the patient's cognitive impairment must be confirmed during the follow-up evaluations. It has not yet been defined how long for is necessary to maintain the cognitive deterioration for the disease progression to be classified as irreversible.

## Biomarkers Associated With Disability Progression

### Imaging Biomarkers: Magnetic Resonance Imaging (MRI)

In progressive MS, the MRI facilitates the demonstration of the substrate of disease progression (i.e., demyelination, axonal degeneration, and microglial activation), predicting thus future disability, and exploring new parameters of treatment efficacy (77). However, the role of MRI in progressive MS regarding diagnostic and monitoring of progression is currently limited (78) (Table 2).

**Table 2:** MRI sequences useful to predict disability and progression in MS.

Sequence*	**Useful findings in impairment prediction and disease progression**	**Technical considerations and limitations**
T2, PD, FLAIR	- Initial lesion load and appearance of new lesions in baseline studies - Topography of lesions (i.e., protuberance, midbrain, and spinal cord)	
DIR, 3D T1, MPRAGE/MP2RAGE	- Greater detection of cortical lesions in baseline study and their increase in number with evolution.	
PSIR	- Superior detection of cortical lesions and their increase in number with evolution. - Higher detection of spinal cord lesions - Detection of spinal cord gray matter involvement.	
3D T1	BRAIN: - Global cerebral atrophy - Cortical atrophy - Deep gray matter atrophy (thalamus/caudate nucleus). - Chronic black holes detection - Slowly expanding lesions (SEL).	- Difficult application in clinical practice: multiple confounding factors can alter its calculation. - Isometric voxels with 1 × 1 × 1 mm resolution - Use of automatic or semiautomatic post-processing tools. - Use of segmentation techniques.
	SPINAL CORD: - Global spinal cord atrophy (measurement in the cervical segment).	- Poor reproducibility
T2* and SWI	- Number of SEL with peripheral iron rim due to the presence of microglia/macrophages	- High magnetic fields (3T and 7T). - Phase imaging
	- Assessment of iron deposition in basal ganglia	- Quantitative evaluation with R2* mapping or QSM
3D-T2-FLAIR post contrast	- Detection of ectopic lymphoid follicles.	- Late acquisition at 10–15 min. - Subtracted images
MTI	Magnetization transfer ratio (MTR): - MTR reduction in ANWM, ANGM, and T2 lesions.	- Used in clinical trials to monitor myelin integrity
Spectroscopy	- Assessment of NAA (N-acetyl-aspartate) levels.	- No conclusive results
DTI	- Alteration of fractional anisotropy (FA) values	- No conclusive results

*PD, Proton Density; FLAIR, fluid-attenuated inversion recovery; DIR, double inversion recovery; MPRAGE, magnetization prepared rapid gradient echo; PSIR, phase-sensitive inversion recovery; SWI, susceptibility-weighted imaging; QSM, quantitative susceptibility mapping; MTI, Magnetization Transfer Imaging; DTI, diffusion tensor imaging*.

#### MRI Findings Associated With Neuroinflammation

The baseline lesion burden in the CNS and the increase in the number of T2 lesions during the follow-up studies are good predictors of both short and long-term cumulative disability (80). The specific localization of T2 lesions in the brain and spinal cord is also crucial in predicting progression. In the early stages of the disease, lesions located in the cerebellum, pons, or midbrain correlate well with a higher risk of future cumulative disability (81). Also, the presence of spinal cord lesions at the initial stages of the RRMS forms has been described as a robust predictor of progression and cumulative disability 15 years after the diagnosis (82).

Slowly expanding lesions (SEL) lie halfway between neuroinflammation and neurodegeneration. They are more frequent in progressive forms of the disease and correspond to active chronic lesions without alteration of the blood-brain barrier. The use of semi-automatic programs to quantify their growth can help with their identification (83). The use of specific sequences permits the identification of iron-laden microglia/macrophages in their periphery (84). The presence of multiple SELs is associated with more aggressive forms of the disease, with shorter intervals between onset and progressive phase of the disease, and a higher motor and cognitive disability (85). In the future, the evaluation of the peripheral edges of the SEL could also be useful to evaluate treatment efficacy (86).

Cortical gray matter lesions are present in all forms of MS but are more frequent in the progressive forms. In the relapsing form of the disease, the basal cortical lesion load and its evolutional accumulation are associated with disability and cognitive impairment at 5 years (87, 88). Spinal cord gray matter involvement is more frequent in PMS patients and is independently associated with disability progression (89).

The presence of asymptomatic gadolinium-enhanced lesions in the basal MRI is associated with shorter time to PMS phase progression and with worse physical and mental impairment (82).

Leptomeningeal infiltrates are markers of inflammation in different MS subtypes and correspond to ectopic lymphoid follicular structures that lead to cortical damage with demyelination and axonal loss. They are more prevalent in the PMS form and correlate well with cortical atrophy and worse disability at 5 years (84). Meningeal enhancement patterns can be variable and depending on the type and persistence over time, there may be a more significant correlation with the disability deterioration (90).

#### MRI Findings Associated With Neurodegeneration

Chronic black holes are hypointense lesions in T1-weighted sequences that persist for more than 6 months (91). The number of black holes at disease onset and its subsequent progression predicts future worsening of disability measured with EDSS (85).

Cerebral atrophy is described in all MS phenotypes, with a progression of the atrophy of 0.5–1.3% per year usually. The cerebral atrophy is one of the objective parameters that have the highest correlation with disease progression at 10 years and correlates with deterioration of the cognitive function (81). Its application in daily clinical practice is difficult due to the multiple confounding factors that can alter its calculation (92). Globally, the level of brain atrophy at disease onset is associated with a more significant future cognitive decline, fatigue, and disability. Finally, the assessment of brain atrophy has been included in clinical trials as a marker of treatment efficacy (85).

Gray matter atrophy is greater in progressive forms of the disease and correlates well with clinical disability. There are differences in the location of the atrophy depending on the clinical form; in SPMS temporal cortex atrophy is more evident (84, 93). Thalamus atrophy in recently diagnosed patients is associated with an increased risk of disability progression and cognitive impairment (78, 84). On the other hand, cortical atrophy of the cerebellar lobes is correlated with cognitive impairment in patients with PMS (78).

Finally, the spinal cord atrophy, measured in the cervical segment, is more significant in the progressive phase and has a strong correlation with disability (94). Furthermore, the evolutional loss of spinal cord volume is an independent marker associated with disease progression (95). Finally, the spinal cord atrophy is a potential biomarker of the efficacy of new drugs but lacks reproducibility across patient populations (94).

#### Advanced MRI Techniques in Clinical Practice

Functional magnetic resonance imaging studies have shown changes in the cortex of patients with PPMS. These changes involve alterations in function of neural networks. The alterations detected could suggest that the cortical adaptation capacity to adapt to tissue damage in patients with progressive forms of MS would also be altered, contributing to functional loss (96).

The Magnetization Transfer Ratio (MTR) is a measurement of tissue integrity. Alteration of its values in apparently normal white matter (ANWM) is more significant in PPMS and SPMS phenotypes. Low initial MTR values in ANWM can predict severe impairment and disability in PPMS. MTR has been used in clinical trials to study tissue remyelination in patients with SPMS (78).

DTI (Diffusion Tensor Imaging) can be used to measure demyelination and axonal loss in T2 lesions, in ANWM and gray matter. The alteration of the anisotropy fraction has been correlated with a physical and cognitive disability (97).

Magnetic resonance spectroscopy studies observed decreased levels of N-acetyl-aspartate (NAA) in both evolving lesions and ANWM and, interestingly, in the cortex of patients with progressive forms (78, 98).

Quantification of iron deposition in basal ganglia has been shown to correlate well with cognitive decline. It has also been proposed as a marker of future cumulative disability in patients with a clinically isolated syndrome (99, 100).

### Neurophysiological Biomarkers

Although the role of neurophysiological tests, as evoked potentials (EP), has been displaced in the last updates of diagnostic criteria in both relapsing and primary progressive MS, there are several studies that have investigated their potential role in predicting disability or even treatment response, and are still a useful paraclinical diagnostic support for MS, enhancing the detection of clinical unapparent demyelinating lesions (101). EP not only assess CNS conductions, being an objective ancillary evaluation of visual, motor (pyramidal), sensory and acoustic pathways, but also can have a glimpse on intracortical processes that are related to the mechanisms of brain plasticity (102).

### Serum and CSF Biomarkers

The search for new biomarkers in multiple sclerosis is necessary for the early diagnosis of the disease, to stratify risk, to predict and monitor progression, to measure treatment response, and to monitor adverse drug effects (103). There are currently a few validated useful biomarkers to predict MS progression, but research in this field has been multiplied in recent years.

One of the essential biomarkers in MS is the oligoclonal bands (OCB). The presence of OCB in the cerebrospinal fluid in patients with a clinically isolated syndrome is an independent predictive factor for moderate disability (EDSS 3.0) in the future (80). Also, intrathecal secretion of IgG has been associated with increased risk of worsening disability in a shorter time (104).

Other CSF molecules have been studied, such as CXCL13 chemokine, which is associated with increased disability in MS patients, but is not specific, and correlates with BOCs (105). The glial fibrillary acidic protein (GFAP) is an intermediate filament protein associated with astrocyte damage and astrogliosis. This protein has been associated with severe disability progression after 8–10 years of disease evolution and is higher in PMS (106, 107). It also correlates with the glycoprotein YKL-40 (CHI3L1), a biomarker associated with progression to EDSS of 6.0 (108).

Neurofilament light chains (NfL) are biomarkers of neuronal damage found in both CSF and serum. In CSF, measured by ELISA, they have been proposed as predictors of severity and disease progression. Higher levels of NfL have been found in patients who switched to the PMS form (109). In a recent prospective study of 607 patients over 12 years in which serum NfL was measured using Simoa assay, a significant relationship was found between EDSS deterioration and NfL serum levels. However, no association was found between serum NFL levels and long-term disease progression in relapsing and between CSF NFL levels and EDSS scores in primary progressive forms (110, 111). These results are contradictory to those found by Barro and Disanto in two different studies, where a correlation was found between high serum NfL levels and long term disability progression in MS (112, 113). A recent meta-analysis of NFL in progressive MS also points out that NFL are more related to acute inflammation rather than being able to differentiate prognosis in progressive MS (114).

### Additional Imaging Biomarkers

Optical coherence tomography (OCT) is an imaging technique that has been used to evaluate retinal integrity in MS patients. Peripapillary retinal nerve fiber layer (pRNFL) and ganglion cell-inner plexiform layer (GCIPL) thickness are recognized as markers of diffuse axonal damage in MS (115, 116).

Few studies have analyzed the role of OCT in patients with progressive forms of MS. Most of these studies are cross-sectional and include heterogeneous populations, in which PMS patients constitute only a small proportion of the studied cohorts.

Other studies demonstrated that pRNFL and GCIPL thinning correlates with a higher EDSS (115, 116). Also, it was proved that the OCT could be useful in predicting disability progression during follow-up. Specifically, the authors of this work demonstrated that presentation with a pRNFL thickness below 88 um confers a higher risk of developing disability after 5 years of follow-up (HR 1.96, 95% CI 1.39–2.76) (117). An independent study also demonstrated that a thickness of RNFL below 88 um is associated with an increased risk of developing cognitive impairment as well, measured by the SDMT (118).

It is worth mentioning that these two studies have been performed in relapsing MS patients, and it is not known whether these results can be extrapolated to progressive forms of the disease.

## Pharmacological Treatment for Progressive Forms

Different drugs have been tested in progressive forms of MS (Table 3). However, most of the clinical trials have shown negative results, probably because of erroneous pharmacological targets, or the strategies used to measure disease progression (79).

**Table 3:** Clinical trials in progressive MS. Adapted from Ciotti JR and Cross AH (79).

**Tested drug**	**Type of MS**	**Number of subjects**	**Duration**	**Primary endpoint**	**Results**
**MECHANISM OF ACTION: NON-SELECTIVE IMMUNOSUPPRESSANTS**					
Cyclophosphamide 750 mg/m^2^ vs. IV glucocorticoids	SPMS	138	2 years	Time to progression (using EDSS)	Failure
Sulfasalazine 500–2,000 mg daily vs. placebo	RRMS, SPMS, PPMS	199	3 years	Time to progression (using EDSS)	Failure
Mitoxantrone 5 or 12 mg/ m^2^ q3 months vs. placebo	PRMS, SPMS	194	1.5 years	Sequentially tested endpoints were change in EDSS, changes in ambulation, relapses, time to first relapse, and changes in SNS	*p* < 0.0001
Cladribine 0.7 or 2.1 mg/kg (total dose over course) vs. placebo	SPMS, PPMS	159	1 year	Mean change in EDSS	Failure
**MECHANISM OF ACTION: IMMUNOMODULATORS**					
IFN beta 1-b 8 million IU every other day vs. placebo (European trial)	SPMS	718	1,5 years	Confirmed progression of disability measured by EDSS	*p* = 0.007
IFN beta 1-b 250 or 160 mcg every other day vs. placebo (American trial)	SPMS	939	3 years	Confirmed progression of disability measured by EDSS	Failure
IFN beta 1-a 22 mcg, 44 mcg vs. placebo (SPECTRIMS)	SPMS	618	3 years	Confirmed progression of disability measured by EDSS	Failure
IFN beta 1-b 8 MUI every other day vs. placebo	PPMS, SPMS	73	2 years	EDSS	Failure
IFN beta 1-a 60 mcg q Weekly vs. placebo (IMPACT)	SPMS	436	2 years	MSFC	*p* = 0.003
Glatiramer acetate 20 mg daily vs. placebo	PPMS	943	3 years	Time to EDSS worsening	Failure
Laquinimod 0.6 mg daily vs. placebo	PPMS	374	1 year	Percentage of change in brain volume	Failure
**MONOCLONAL ANTIBODY**					
Rituximab 1,000 mg q6 months vs. placebo	PPMS	439	2 years	Time to EDSS worsening	Failure
Natalizumab 300 mg IV q4 weeks vs. placebo	SPMS	889	2 years	Percentage of patients with progression in EDSS, T25FW or 9HPT	Failure
Ocrelizumab 600 mg q6 months vs. placebo	PPMS	732	3 years	Percentage of patients with progression in EDSS	*p* = 0.03
Opicinumab 3 or 10 or 30 or 100 mg/kg every 4 weeks + IFN beta 1-a vs. placebo +IFN beta 1a	RRMS, active SPMS	418	1.5 years	Percentage of patients with improvements in EDSS, T25FW, 9HPT o PASAT	Failure
**MECHANISM OF ACTION: SELECTIVE IMMUNOSUPPRESSANTS**					
Siponimod 0.25-2 mg vs. placebo	SPMS	1651	3 years	Confirmed progression of disability measured by EDSS	*p* = 0.013
Fingolimod 0.5 mg or 1.25 mg daily vs. placebo	PPMS	970	5 years	Time to progression measured by EDSS, T25FW, or 9HPT	Failure
**MECHANISM OF ACTION: NEUROPROTECTOR**					
Ibudilast 100 mg daily vs. placebo (added to patient's immunomodulator treatment)	SPMS, PPMS	255	2 years	Change in brain volume assessed by BPF	*p* = 0.04
Biotin 300 mg daily vs. placebo (added to patient's immunomodulator treatment)	SPMS, PPMS	154	1 year	Proportion of disability improvement (EDSS and T25FW)	*p* = 0.005

*9HPT, Nine-hole peg test; BPF, Brain parenchymal fraction; EDSS, expanded disability Status score; IU, international units; MSFC, Multiple sclerosis functional composite; PPMS, primary progressive multiple sclerosis; PRMS, progressive relapsing multiple sclerosis; RRMS, relapsing-remitting multiple sclerosis; SNS, standardized neurological status; SPMS, secondary progressive multiple sclerosis; T25FW, timed 25-foot walk*.

### Why Do Most Therapeutic Strategies Fail in Progressive Forms of MS?

The main reason that the majority of drugs tested so far failed to control the progressive phase of the disease is because of the inability to reverse the pathophysiological mechanism responsible for progression (119). The therapeutic options available for progressive MS are insufficient, which continues to be a significant challenge for researchers (120). In PMS, it was stated that inflammation might be partly compartmentalized in the brain, and the intact blood-brain barrier prevents the migration of the drugs into the CNS, limiting thus their effect (120).

The SPMS form may still be associated with slight clinical disease activity. On the other hand, the effects that most of the current disease-modifying treatments (DMT) exert are predominately on the inflammatory phase of the disease (121). Hence, in clinical trials of PMS, some treatments reached their best results in the subgroups of patients with slight disease activity or shorter times of evolution (122–124). Table 4 shows the currently approved treatments for PMS.

**Table 4:** Approved treatments for progressive MS (125–129).

**Drugs**	**Indications approved by European Medicines Agency**
Interferon beta 1-b sc Interferon beta 1-a sc	Relapsing SPMS
Mitoxantrone	Highly active relapsing MS, associated with a rapid evolution of disability in which there are no alternative therapeutic options.
Ocrelizumab	Active PPMS Relapsing SPMS
Siponimod	Recently FDA and EMA approved for active SPMS

*EMA, European Medicines Agency; FDA, Food and Drugs Administration; SPMS, secondary progressive multiple sclerosis; sc, subcutaneous; MS, multiple sclerosis*.

There are many drugs that have failed in their clinical trials in the treatment of PMS, and their treatment continues to be a challenge today (120). The causes of these failures are diverse.

The most obvious cause, as it is mentioned before, is that the drugs used in clinical trials have not been able to reverse or to stop the pathogenic mechanisms of the disease (119). In PMS, inflammation might be partly compartmentalized within the CNS, and the intact blood-brain barrier prevents the migration of drugs into the CNS, limiting thus their effect (120). Sometimes the criteria for deciding that a molecule could work in a PMS clinical trial is its success in a previous clinical trial in relapsing forms of MS, and this may not be extrapolated to PMS (46).

Other reason at stake could be an inappropriate clinical trial design, outcome, sample size or population. As an example, interferon beta in SPMS has inconsistently showed efficacy for preventing progression in patients with SPMS. These inconsistencies between clinical trials seem to be mostly related to differences in previous inflammatory disease activity between populations, rather to a real effect over the degenerative process. A systematic review of the clinical trials in SPMS pointed out to younger patients and those with pre-treatment relapses having better outcomes than older patients with longer disease duration and those who did not experience pre-study relapses (130). On the other side, two trials with interferon beta failed to show a reduction in disability progression in PPMS patients (131, 132). However, the trial populations were too small to allow definitive conclusions on the efficacy of interferon beta treatment in patients with PPMS (133).

Another example applies to the trial of fingolimod for PPMS, in which more than 40% of patients were 50 or more years of age, and more than 85% had no contrast-enhanced lesions (134). The tools for measuring progression may not be sensitive enough to detect progression, the primary objectives may not be the most appropriate, as well as the length of the trials, too short for PMS (135). In the ASCEND trial (natalizumab vs. placebo in SPMS), the results were negative for the primary endpoint and for the T25FW as secondary endpoint. However, an effect on the 9HPT was observed as secondary outcome (136). This latter study in SPMS contrasts with the moderate but positive effect of siponimod, a selective sphingosine-1-phosphate receptors 1 and 5 modulator, on disability progression in SPMS. As in the ASCEND trial, the clinical benefit of siponimod was more evident in upper extremity function (9HPT) than in lower extremity function (T25FW) (122).

The failure of the OLYMPUS trial with rituximab (124) can be partly attributed to a miscalculation of the sample size, probably due to a false expectation of progression rate from the placebo group. This highlights the importance of adequate planning prior to the study and of gathering more information to infer the probability of progression in the placebo group. The ORATORIO design was able to overcome these problems that arose in the OLYMPUS clinical trial (133, 137). Long-term effects of ocrelizumab in PPMS have been observed with sustained benefit through 6.5 years of follow-up (138).

### Therapeutic Failure in Progressive Forms

There is no consensus on the definition of therapeutic failure in progressive forms of the MS. Persistence or appearance of new activity, either radiological or clinical, should be considered as a therapeutic failure. However, in the absence of inflammatory activity, measuring progression can be more complex. In PMS, EDSS has limited value in capturing slight changes in clinical status, and should probably be complemented by other scales such as T25FW and 9HPT. An alternative option might be to consider the No Evidence of Progression or Active Disease (NEPAD), which is a combined outcome measure used in a clinical trial in patients with PPMS. It includes the absence of progression and takes into account relapses and MRI activity, which can both be present during the progressive phase (50).

On the other hand, DMT modulate the evolution of the disease. However, we cannot confirm that they can stop its progression. Thus, it is expected that patient will progress during the disease course over time, despite responding to DMT at disease onset. This point leads to another question: should we maintain treatments indefinitely or should we stop it after a certain level of disability is reached? In this respect, the American Academy of Neurology guidelines suggest to consider withdrawing DMT in patients with EDSS ≥ 7.0 in the absence of clinical or radiological activity (139), although this proposal is pending validation.

## Conclusions

The progression prevention of irreversible disability is the primary therapeutic objective for every neurologist in the management of patients with MS. Despite this, the definition of progression remains controversial, as well as its clinical and radiological identification in daily practice.

The clinical definitions of disability progression are based on the increase in EDSS over a specific time, making it necessarily retrospective. This means that nowadays, early identification of progression is challenging, but it would be desirable to take useful therapeutic actions for the patients. The different examination scales used in clinical practice, despite having significant advantages, can be deficient if used in isolation. Due to the multidimensional characteristics of the disease, composite measures, such as EDSS plus, could precisely identify progression as quickly as possible.

The search for biomarkers that help identify progression in MS is essential. MRI mainly offers prognostic factors but does not allow us to identify progression when it occurs. There are few validated biomarkers available for MS progression. Research in this field has been multiplied in recent years.

Pharmacotherapy in progressive forms is currently very limited. The pathophysiology of the disease may be responsible for the repeated failure of various molecules to prevent progression. Pharmacotherapy, along with the early identification of progression, is one of the significant challenges in progressive multiple sclerosis.

## Author Contributions

VM-L and FP-M conceptualized, designed and supervised the review, contributed the specific topics, revised the manuscript for intellectual content, and acted as co-coordinators. LB-R, JC-P, SE-M, JF-L, LF, YH, NT, and AV-J contributed the specific topics and assisted in drafting and revising the final version of the manuscript. All authors read and approved the final manuscript.

## Conflict of Interest

VM-L has received compensation for consulting services and speaking honoraria from Almirall, Biogen, Genzyme, Merck Serono, Novartis, Roche, Terumo, Sanofi and Teva. LB-R has received compensation for consulting services and speaking honoraria from Biogen, Sanofy-Genzyme, Merck Serono, Novartis, Roche, and Teva. JC-P has received compensation for consulting services from Roche. SE-M has received compensation for consulting services and speaking honoraria from Biogen Idec, Novartis, Merck, Bayer, Sanofi-Genzyme, Roche, and Teva. JF-L has received compensation for consulting services from Roche. LF has received compensation for consulting services from Roche, Merck, Novartis and Genzyme, for speaking honoraria from Roche, Merck and Novartis, and for traveling grants from Genzyme, Roche and Novartis. YH has received compensation for consulting services from Roche and Merck, Novartis, Teva and Genzyme, for speaking, honoraria from Roche, Merck and Novartis, and for traveling grants from Merck, Genzyme, Roche and Novartis. NT has received compensation for consulting services, traveling grants and speaking honoraria from Bayer Schering Pharma, Biogen Idec, Merck Serono, Novartis, Sanofi-Aventis, Teva, and Roche. AV-J has received investigation grants Juan Rodes (JR16/00024) from Fondo de Investigaciones Sanitarias, Instituto de Salud Carlos III, and has received compensation for consulting services, participation in advisory boards, and speaking honoraria from Novartis, Stendhal, Roche, Teva, Biogen, and Genzyme-Sanofi. FP-M has received compensation for consulting services and speaking honoraria from Roche, Sanofi-Genzyme y Biogen, and speaking honoraria from Novartis, Almirall and Teva.

## References

[1] Confavreux C Vukusic S Adeleine P. Early clinical predictors and progression of irreversible disability in multiple sclerosis: an amnesic process. Brain. (2003) 126:770–82. 10.1093/brain/awg081 12615637

[2] Leray E Yaouanq J Le Page E Coustans M Laplaud D Oger J. Evidence for a two-stage disability progression in multiple sclerosis. Brain. (2010) 133:1900–13. 10.1093/brain/awq076 20423930 PMC2892936

[3] Scalfari A Lederer C Daumer M Nicholas R Ebers G Muraro P. The relationship of age with the clinical phenotype in multiple sclerosis. Mult Scler. (2016) 22:1750–8. 10.1177/1352458516630396 26869531

[4] Coret F Pérez-Miralles FC Gascón F Alcalá C Navarré A Bernad A. Onset of secondary progressive multiple sclerosis is not influenced by current relapsing multiple sclerosis therapies. Mult Scler J Exp Transl Clin. (2018) 4:2055217318783347. 10.1177/2055217318783347 30090637 PMC6077906

[5] Lublin FD Reingold SC. Defining the clinical course of multiple sclerosis: results of an international survey. National Multiple Sclerosis Society (USA) Advisory Committee on Clinical Trials of New Agents in Multiple Sclerosis. Neurology. (1996) 46:907–11. 10.1212/WNL.46.4.907 8780061

[6] Lublin FD Reingold SC Cohen JA Cutter GR Sorensen PS Thompson AJ. Defining the clinical course of multiple sclerosis: The 2013 revisions. Neurology. (2014) 83:278–86. 10.1212/WNL.0000000000000560 24871874 PMC4117366

[7] Zeydan B Kantarci OH. Progressive forms of multiple sclerosis: distinct entity or age-dependent phenomena. Neurol Clin. (2018) 36:163–71. 10.1016/j.ncl.2017.08.006 29157397

[8] Petruzzo M Reia A Maniscalco GT Luiso F Lanzillo R Russo CV. The Framingham cardiovascular risk score and 5-year progression of multiple sclerosis. Eur J Neurol. (2021) 28:893–900. 10.1111/ene.14608 33091222

[9] Ostolaza A Corroza J Ayuso T. Multiple sclerosis and aging: comorbidity and treatment challenges. Mult Scler Relat Disord. (2021) 50:102815. 10.1016/j.msard.2021.102815 33581613

[10] Cisternas M Bartolome L Gitar B Hulbert E Trenz H Patel V. Health care resource utilization and disease modifying treatment use in multiple sclerosis patients by age and insurance type. Curr Med Res Opin. (2021). 10.1080/03007995.2021.1885367. [Epub ahead of print]. 33535846

[11] Schumacker GA Beebe G Kibler RF Kurland LT Kurtzke JF McDowell F. Problems of experimental trials of therapy in multiple sclerosis: report by the panel on the evaluation of experimental trials of therapy in multiple sclerosis. Ann N Y Acad Sci. (1965) 122:552–68. 10.1111/j.1749-6632.1965.tb20235.x 14313512

[12] Poser CM Paty DW Scheinberg L McDonald WI Davis FA Ebers GC. New diagnostic criteria for multiple sclerosis: guidelines for research protocols. Ann Neurol. (1983) 13:227–31. 10.1002/ana.410130302 6847134

[13] Thompson AJ Kermode AG Wicks D MacManus DG Kendall BE Kingsley DPE. Major differences in the dynamics of primary and secondary progressive multiple sclerosis. Ann Neurol. (1991) 29:53–62. 10.1002/ana.410290111 1996879

[14] McDonald WI Compston A Edan G Goodkin D Hartung HP Lublin FD. Recommended diagnostic criteria for multiple sclerosis: guidelines from the International Panel on the diagnosis of multiple sclerosis. Ann Neurol. (2001) 50:121–7. 10.1002/ana.1032 11456302

[15] Polman CH Reingold SC Edan G Filippi M Hartung H-P Kappos L. Diagnostic criteria for multiple sclerosis: 2005 revisions to the “McDonald Criteria”. Ann Neurol. (2005) 58:840–6. 10.1002/ana.20703 16283615

[16] Polman CH Reingold SC Banwell B Clanet M Cohen J a. Filippi M. Diagnostic criteria for multiple sclerosis: 2010 Revisions to the McDonald criteria. Ann Neurol. (2011) 69:292–302. 10.1002/ana.22366 21387374 PMC3084507

[17] Thompson AJ Banwell BL Barkhof F Carroll WM Coetzee T Comi G. Diagnosis of multiple sclerosis: 2017 revisions of the McDonald criteria. Lancet Neurol. (2018) 17:162–73. 10.1016/S1474-4422(17)30470-2 29275977

[18] Kalincik T Cutter G Spelman T Jokubaitis V Havrdova E Horakova D. Defining reliable disability outcomes in multiple sclerosis. Brain. (2015) 138:3287–98. 10.1093/brain/awv258 26359291

[19] Lassmann H. Multiple Sclerosis Pathology. Cold Spring Harb Perspect Med. (2018) 8:a028936. 10.1101/cshperspect.a028936 29358320 PMC5830904

[20] Haider L Zrzavy T Hametner S Höftberger R Bagnato F Grabner G. The topograpy of demyelination and neurodegeneration in the multiple sclerosis brain Brain Advance Access. Brain. (2016) 139:807–15. 10.1093/brain/awv398 26912645 PMC4766379

[21] Lucchinetti C Brück W Parisi J Scheithauer B Rodriguez M Lassmann H. Heterogeneity of multiple sclerosis lesions: implications for the pathogenesis of demyelination. Ann Neurol. (2000) 47:707–17. 10.1002/1531-8249(200006)47:6<707::AID-ANA3>3.0.CO;2-Q 10852536

[22] Metz I Weigand SD Popescu BFG Frischer JM Parisi JE Guo Y. Pathologic heterogeneity persists in early active multiple sclerosis lesions. Ann Neurol. (2014) 75:728–38. 10.1002/ana.24163 24771535 PMC4070313

[23] Barnett MH Prineas JW. Relapsing and remitting multiple sclerosis: Pathology of the newly forming lesion. Ann Neurol. (2004) 55:458–68. 10.1002/ana.20016 15048884

[24] Breij ECW Brink BP Veerhuis R van den Berg C Vloet R Yan R. Homogeneity of active demyelinating lesions in established multiple sclerosis. Ann Neurol. (2008) 63:16–25. 10.1002/ana.21311 18232012

[25] Kutzelnigg A Lucchinetti CF Stadelmann C Brück W Rauschka H Bergmann M. Cortical demyelination and diffuse white matter injury in multiple sclerosis. Brain. (2005) 128:2705–12. 10.1093/brain/awh641 16230320

[26] Peterson JW Bö L Mörk S Chang A Trapp BD. Transected neurites, apoptotic neurons, and reduced inflammation in cortical multiple sclerosis lesions. Ann Neurol. (2001) 50:389–400. 10.1002/ana.1123 11558796

[27] Fischer MT Wimmer I Höftberger R Gerlach S Haider L Zrzavy T. Disease-specific molecular events in cortical multiple sclerosis lesions. Brain. (2013) 136:1799–815. 10.1093/brain/awt110 23687122 PMC3673462

[28] Mahad D Ziabreva I Lassmann H Turnbull D. Mitochondrial defects in acute multiple sclerosis lesions. Brain. (2008) 131:1722–35. 10.1093/brain/awn105 18515320 PMC2442422

[29] Trapp BD Stys PK. Virtual hypoxia and chronic necrosis of demyelinated axons in multiple sclerosis. Lancet Neurol. (2009) 8:280–91. 10.1016/S1474-4422(09)70043-2 19233038

[30] Lassmann H van Horssen J Mahad D. Progressive multiple sclerosis: pathology and pathogenesis. Nat Rev Neurol. (2012) 8:647–56. 10.1038/nrneurol.2012.168 23007702

[31] Kurtzke JF. Rating neurologic impairment in multiple sclerosis: an expanded disability status scale (EDSS). Neurology. (1983) 33:1444–52. 10.1212/WNL.33.11.1444 6685237

[32] European Medicines Agency. Clinical Investigation of Medicinal Products for the Treatment of Multiple Sclerosis (2015). Available online at: https://www.ema.europa.eu/en/documents/scientific-guideline/guideline-clinical-investigation-medicinal-products-treatment-multiple-sclerosis_en-0.pdf (accessed September 20, 2020).

[33] Lorscheider J Buzzard K Jokubaitis V Spelman T Havrdova E Horakova D. Defining secondary progressive multiple sclerosis. Brain. (2016) 139:2395–405. 10.1093/brain/aww173 27401521

[34] Weinshenker BG Issa M Baskerville J. Meta-analysis of the placebo-treated groups in clinical trials of progressive MS. Neurology. (1996) 46:1613–9. 10.1212/WNL.46.6.1613 8649559

[35] Rudick RA Kappos L. Measuring disability in relapsing-remitting MS. Neurology. (2010) 75:296–7. 10.1212/WNL.0b013e3181ecf815 20592252

[36] Roosendaal SD Schoonheim MM Hulst HE Sanz-Arigita EJ Smith SM Geurts JJG. Resting state networks change in clinically isolated syndrome. Brain. (2010) 133:1612–21. 10.1093/brain/awq058 20356855

[37] Paz Soldan MM Novotna M Abou Zeid N Kale N Tutuncu M Crusan DJ. Relapses and disability accumulation in progressive multiple sclerosis. Neurology. (2015) 84:81–8. 10.1212/WNL.0000000000001094 25398229 PMC4336097

[38] Cree BAC Hollenbach JA Bove R Kirkish G Sacco S Caverzasi E. Silent progression in disease activity-free relapsing multiple sclerosis. Ann Neurol. (2019) 85:653–66. 10.1002/ana.25463 30851128 PMC6518998

[39] Gil-Perotin S Alcalá C Pérez-Miralles FC Casanova B. Silent progression or bout onset progressive multiple sclerosis? Ann Neurol. (2019) 86:472–2. 10.1002/ana.25537 31251817

[40] Ebers GC Heigenhauser L Daumer M Lederer C Noseworthy JH. Disability as an outcome in MS clinical trials. Neurology. (2008) 71:624–31. 10.1212/01.wnl.0000313034.46883.16 18480462

[41] Amato MP Portaccio E. Clinical outcome measures in multiple sclerosis. J Neurol Sci. (2007) 259:118–122. 10.1016/j.jns.2006.06.031 17376487

[42] Hirst CL Ingram G Pickersgill TP Robertson NP. Temporal evolution of remission following multiple sclerosis relapse and predictors of outcome. Mult Scler. (2012) 18:1152–8. 10.1177/1352458511433919 22217582

[43] Kappos L Butzkueven H Wiendl H Spelman T Pellegrini F Chen Y. Greater sensitivity to multiple sclerosis disability worsening and progression events using a roving versus a fixed reference value in a prospective cohort study. Mult Scler. (2018) 24:963–73. 10.1177/1352458517709619 28554238 PMC6029149

[44] Lechner-Scott J Brunnschweiler H Kappos L Committee TSS. Is it possible to achieve cross-cultural european agreement in the assessment of neurological deficits? First experiences in the european interferon-beta 1B trial for secondary progressive MS. J Neuroimmunol. (1995) 56-63:42. 10.1016/0165-5728(95)98993-L

[45] Cutter GR Baier ML Rudick RA Cookfair DL Fischer JS Petkau J. Development of a multiple sclerosis functional composite as a clinical trial outcome measure. Brain. (1999) 122:871–82. 10.1093/brain/122.5.871 10355672

[46] Ontaneda D Fox RJ Chataway J. Clinical trials in progressive multiple sclerosis: lessons learned and future perspectives. Lancet Neurol. (2015) 14:208–23. 10.1016/S1474-4422(14)70264-9 25772899 PMC4361791

[47] Bosma LVAE Kragt JJ Brieva L Khaleeli Z Montalban X Polman CH. Progression on the multiple sclerosis functional composite in multiple sclerosis: what is the optimal cut-off for the three components? Mult Scler. (2010) 16:862–7. 10.1177/1352458510370464 20488826

[48] Hoogervorst ELJ Kalkers NF Uitdehaag BMJ Polman CH. A study validating changes in the multiple sclerosis functional composite. Arch Neurol. (2002) 59:113–6. 10.1001/archneur.59.1.113 11790238

[49] Cadavid D Cohen JA Freedman MS Goldman MD Hartung H-P Havrdova E. The EDSS-Plus, an improved endpoint for disability progression in secondary progressive multiple sclerosis. Mult Scler. (2017) 23:94–105. 10.1177/1352458516638941 27003945

[50] Wolinsky JS Montalban X Hauser SL Giovannoni G Vermersch P Bernasconi C. Evaluation of no evidence of progression or active disease (NEPAD) in patients with primary progressive multiple sclerosis in the ORATORIO trial. Ann Neurol. (2018) 84:527–36. 10.1002/ana.25313 30155979 PMC6220799

[51] Motl RW Cohen JA Benedict R Phillips G LaRocca N Hudson LD. Validity of the timed 25-foot walk as an ambulatory performance outcome measure for multiple sclerosis. Mult Scler. (2017) 23:704–10. 10.1177/1352458517690823 28206828 PMC5405807

[52] Feys P Lamers I Francis G Benedict R Phillips G LaRocca N. The Nine-Hole Peg Test as a manual dexterity performance measure for multiple sclerosis. Mult Scler. (2017) 23:711–20. 10.1177/1352458517690824 28206826 PMC5405844

[53] Balcer LJ Raynowska J Nolan R Galetta SL Kapoor R Benedict R. Validity of low-contrast letter acuity as a visual performance outcome measure for multiple sclerosis. Mult Scler. (2017) 23:734–47. 10.1177/1352458517690822 28206829 PMC5407511

[54] Sola-Valls N Blanco Y Sepúlveda M Llufriu S Martínez-Lapiscina EH La Puma D. Walking function in clinical monitoring of multiple sclerosis by telemedicine. J Neurol. (2015) 262:1706–13. 10.1007/s00415-015-7764-x 25957639

[55] Block VJ Bove R Zhao C Garcha P Graves J Romeo AR. Association of Continuous Assessment Of Step Count By Remote Monitoring With Disability Progression Among Adults With Multiple Sclerosis. JAMA Netw Open. (2019) 2:e190570. 10.1001/jamanetworkopen.2019.0570 30874777 PMC6484622

[56] Dawson J Doll H Fitzpatrick R Jenkinson C Carr AJ. The routine use of patient reported outcome measures in healthcare settings. BMJ. (2010) 340:c186. 10.1136/bmj.c186 20083546

[57] Zhang Y Taylor BV Simpson S Blizzard L van der Mei I. Patient-reported outcomes are worse for progressive-onset multiple sclerosis than relapse-onset multiple sclerosis, particularly early in the disease process. Eur J Neurol. (2019) 26:155–61. 10.1111/ene.13786 30133059

[58] Khurana V Sharma H Afroz N Callan A Medin J. Patient-reported outcomes in multiple sclerosis: a systematic comparison of available measures. Eur J Neurol. (2017) 24:1099–7. 10.1111/ene.13339 28695634

[59] Jenkinson C Coulter A Wright L. Short form 36 (SF36) health survey questionnaire: normative data for adults of working age. BMJ. (1993) 306:1437–440. 10.1136/bmj.306.6890.1437 8518639 PMC1677870

[60] Prevolnik Rupel V Divjak M Zrubka Z Rencz F Gulácsi L Golicki D. EQ-5D studies in nervous system diseases in eight Central and East European countries: a systematic literature review. Eur J Heal Econ. (2019) 20:109–17. 10.1007/s10198-019-01068-9 31098882

[61] Simeoni M Auquier P Fernandez O Flachenecker P Stecchi S Constantinescu C. Validation of the multiple sclerosis international quality of life questionnaire. Mult Scler. (2008) 14:219–30. 10.1177/1352458507080733 17942521

[62] Hobart J Lamping D Fitzpatrick R Riazi A Thompson A. The Multiple Sclerosis Impact Scale (MSIS-29): a new patient-based outcome measure. Brain. (2001) 124:962–73. 10.1093/brain/124.5.962 11335698

[63] Hobart JC Riazi A Lamping DL Fitzpatrick R Thompson AJ. Measuring the impact of MS on walking ability: the 12-Item MS Walking Scale (MSWS-12). Neurology. (2003) 60:31–6. 10.1212/WNL.60.1.31 12525714

[64] Kos D Kerckhofs E Carrea I Verza R Ramos M Jansa J. Evaluation of the modified fatigue impact scale in four different European countries. Mult Scler. (2005) 11:76–80. 10.1191/1352458505ms1117oa 15732270

[65] Kalb R Beier M Benedict RH Charvet L Costello K Feinstein A. Recommendations for cognitive screening and management in multiple sclerosis care. Mult Scler. (2018) 24:1665–80. 10.1177/1352458518803785 30303036 PMC6238181

[66] Amato MP Morra VB Falautano M Ghezzi A Goretti B Patti F. Cognitive assessment in multiple sclerosis-an Italian consensus. Neurol Sci. (2018) 39:1317–24. 10.1007/s10072-018-3427-x 29766386

[67] Amato MP Prestipino E Bellinvia A. Identifying risk factors for cognitive issues in multiple sclerosis. Expert Rev Neurother. (2019) 19:333–47. 10.1080/14737175.2019.1590199 30829076

[68] Dekker I Eijlers AJC Popescu V Balk LJ Vrenken H Wattjes MP. Predicting clinical progression in multiple sclerosis after 6 and 12 years. Eur J Neurol. (2019) 26:893–902. 10.1111/ene.13904 30629788 PMC6590122

[69] Planche V Gibelin M Cregut D Pereira B Clavelou P. Cognitive impairment in a population-based study of patients with multiple sclerosis: differences between late relapsing-remitting, secondary progressive and primary progressive multiple sclerosis. Eur J Neurol. (2016) 23:282–9. 10.1111/ene.12715 25903918

[70] Sumowski JF Benedict R Enzinger C Filippi M Geurts JJ Hamalainen P. Cognition in multiple sclerosis: State of the field and priorities for the future. Neurology. (2018) 90:278–88. 10.1212/WNL.0000000000004977 29343470 PMC5818015

[71] Morrow SA Drake A Zivadinov R Munschauer F Weinstock-Guttman B Benedict RHB. Predicting loss of employment over three years in multiple sclerosis: clinically meaningful cognitive decline. Clin Neuropsychol. (2010) 24:1131–45. 10.1080/13854046.2010.511272 20830649

[72] Moccia M Lanzillo R Palladino R Chang KC-M Costabile T Russo C. Cognitive impairment at diagnosis predicts 10-year multiple sclerosis progression. Mult Scler. (2016) 22:659–67. 10.1177/1352458515599075 26362896

[73] Deloire M Ruet A Hamel D Bonnet M Brochet B. Early cognitive impairment in multiple sclerosis predicts disability outcome several years later. Mult Scler. (2010) 16:581–7. 10.1177/1352458510362819 20194578

[74] Christodoulou C Melville P Scherl WF Macallister WS Abensur RL Troxell RM. Negative affect predicts subsequent cognitive change in multiple sclerosis. J Int Neuropsychol Soc. (2009) 15:53–61. 10.1017/S135561770809005X 19128528

[75] Debouverie M Pittion-Vouyovitch S Louis S Guillemin F. Natural history of multiple sclerosis in a population-based cohort. Eur J Neurol. (2008) 15:916–21. 10.1111/j.1468-1331.2008.02241.x 18637953

[76] Pardini M Uccelli A Grafman J Yaldizli Ö Mancardi G Roccatagliata L. Isolated cognitive relapses in multiple sclerosis. J Neurol Neurosurg Psychiatry. (2014) 85:1035–7. 10.1136/jnnp-2013-307275 24686566

[77] Filippi M Preziosa P Rocca MA. MRI in multiple sclerosis: what is changing? Curr Opin Neurol. (2018) 31:386–95. 10.1097/WCO.0000000000000572 29952834

[78] Petracca M Margoni M Bommarito G Inglese M. Monitoring progressive multiple sclerosis with novel imaging techniques. Neurol Ther. (2018) 7:265–85. 10.1007/s40120-018-0103-2 29956263 PMC6283788

[79] Ciotti JR Cross AH. Disease-modifying treatment in progressive multiple sclerosis. Curr Treat Options Neurol. (2018) 20:12. 10.1007/s11940-018-0496-3 29627873

[80] Tintore M Rovira A. Rio J Otero-Romero S Arrambide G Tur C. Defining high, medium and low impact prognostic factors for developing multiple sclerosis. Brain. (2015) 138:1863–74. 10.1093/brain/awv105 25902415

[81] Wattjes MP Rovira À Miller D Yousry TA Sormani MP de Stefano MP. MAGNIMS consensus guidelines on the use of MRI in multiple sclerosis-establishing disease prognosis and monitoring patients. Nat Rev Neurol. (2015) 11:597–606. 10.1038/nrneurol.2015.157 26369511

[82] Brownlee WJ Altmann DR Prados F Miszkiel KA Eshaghi A Gandini Wheeler-Kingshott CAM. Early imaging predictors of long-term outcomes in relapse-onset multiple sclerosis. Brain. (2019) 142:2276–87. 10.1093/brain/awz156 31342055

[83] Elliott C Wolinsky JS Hauser SL Kappos L Barkhof F Bernasconi C. Slowly expanding/evolving lesions as a magnetic resonance imaging marker of chronic active multiple sclerosis lesions. Mult Scler. (2019) 25:1915–25. 10.1177/1352458518814117 30566027 PMC6876256

[84] Cortese R Collorone S Ciccarelli O Toosy AT. Advances in brain imaging in multiple sclerosis. Ther Adv Neurol Disord. (2019) 12:1756286419859722. 10.1177/1756286419859722 31275430 PMC6598314

[85] Kaunzner UW Gauthier SA. MRI in the assessment and monitoring of multiple sclerosis: an update on best practice. Ther Adv Neurol Disord. (2017) 10:247–61. 10.1177/1756285617708911 28607577 PMC5453402

[86] Absinta M Sati P Masuzzo F Nair G Sethi V Kolb H. Association of chronic active multiple sclerosis lesions with disability in vivo. JAMA Neurol. (2019) 76:1474–83. 10.1001/jamaneurol.2019.2399 31403674 PMC6692692

[87] Calabrese M Rocca MA Atzori M Mattisi I Favaretto A Perini P. A 3-year magnetic resonance imaging study of cortical lesions in relapse-onset multiple sclerosis. Ann Neurol. (2010) 67:376–83. 10.1002/ana.21906 20373349

[88] Calabrese M Poretto V Favaretto A Alessio S Bernardi V Romualdi C. Cortical lesion load associates with progression of disability in multiple sclerosis. Brain. (2012) 135:2952–61. 10.1093/brain/aws246 23065788

[89] Kearney H Miszkiel KA Yiannakas MC Altmann DR Ciccarelli O Miller DH. Grey matter involvement by focal cervical spinal cord lesions is associated with progressive multiple sclerosis. Mult Scler. (2016) 22:910–20. 10.1177/1352458515604905 26432854

[90] Jonas SN Izbudak I Frazier AA Harrison DM. Longitudinal persistence of meningeal enhancement on postcontrast 7T 3D-FLAIR MRI in multiple sclerosis. AJNR Am J Neuroradiol. (2018) 39:1799–805. 10.3174/ajnr.A5796 30213813 PMC6177296

[91] Dupuy SL Tauhid S Kim G Chu R Tummala S Hurwitz S. MRI detection of hypointense brain lesions in patients with multiple sclerosis: T1 spin-echo vs. gradient-echo. Eur J Radiol. (2015) 84:1564–68. 10.1016/j.ejrad.2015.05.004 26044294

[92] Steenwijk MD Geurts JJG Daams M Tijms BM Wink AM Balk LJ. Cortical atrophy patterns in multiple sclerosis are non-random and clinically relevant. Brain. (2015) 139:115–26. 10.1093/brain/awv337 26637488

[93] Sastre-Garriga J Pareto D Rovira À. Brain atrophy in multiple sclerosis: clinical relevance and technical aspects. Neuroimaging Clin N Am. (2017) 27:289–300. 10.1016/j.nic.2017.01.002 28391787

[94] Casserly C Seyman EE Alcaide-Leon P Guenette M Lyons C Sankar S. Spinal cord atrophy in multiple sclerosis: a systematic review and meta-analysis. J Neuroimaging. (2018) 28:556–86. 10.1111/jon.12553 30102003

[95] Lukas C Knol DL Sombekke MH Bellenberg B Hahn HK Popescu V. Cervical spinal cord volume loss is related to clinical disability progression in multiple sclerosis. J Neurol Neurosurg Psychiatry. (2015) 86:410–18. 10.1136/jnnp-2014-308021 24973341

[96] Rocca MA Absinta M Filippi M. The role of advanced magnetic resonance imaging techniques in primary progressive MS. J Neurol. (2012) 259:611–21. 10.1007/s00415-011-6195-6 21814822

[97] Fernandez O Martin R Rovira A Llufriu S Vidal-Jordana A Fernandez-Sanchez VE. Biomarkers in multiple sclerosis: an update for 2014. Rev Neurol. (2014) 58:553–70. 10.33588/rn.5812.2014247 24915032

[98] Narayana PA. Magnetic resonance spectroscopy in the monitoring of multiple sclerosis. J Neuroimaging. (2005) 15:46S. 10.1177/1051228405284200 16385018 PMC1351238

[99] Fujiwara E Kmech JA Cobzas D Sun H Seres P Blevins G. Cognitive implications of deep gray matter iron in multiple sclerosis. AJNR Am J Neuroradiol. (2017) 38:942–8. 10.3174/ajnr.A5109 28232497 PMC7960387

[100] Langkammer C Liu T Khalil M Enzinger C Jehna M Fuchs S. Quantitative susceptibility mapping in multiple sclerosis. Radiology. (2013) 267:551–9. 10.1148/radiol.12120707 23315661 PMC3632806

[101] Aminoff MJ. Electrophysiologic evaluation of patients with multiple sclerosis. Neurol Clin. (1985) 3:663–74. 10.1016/S0733-8619(18)31028-4 3900686

[102] Ferrazzano G Crisafulli SG Baione V Tartaglia M Cortese A Frontoni M. Early diagnosis of secondary progressive multiple sclerosis: focus on fluid and neurophysiological biomarkers. J Neurol. (2020) 10.1007/s00415-020-09964-4 32504180

[103] Comabella M Montalban X. Body fluid biomarkers in multiple sclerosis. Lancet Neurol. (2014) 13:113–26. 10.1016/S1474-4422(13)70233-3 24331797

[104] Gasperi C Salmen A Antony G Bayas A Heesen C Kümpfel T. Association of intrathecal immunoglobulin g synthesis with disability worsening in multiple sclerosis. JAMA Neurol. (2019) 76:841–9. 10.1001/jamaneurol.2019.0905 31034002 PMC6583696

[105] Khademi M Kockum I Andersson ML Iacobaeus E Brundin L Sellebjerg F. Cerebrospinal fluid CXCL13 in multiple sclerosis: a suggestive prognostic marker for the disease course. Mult Scler. (2011) 17:335–43. 10.1177/1352458510389102 21135023

[106] Petzold A Eikelenboom MJ Gveric D Keir G Chapman M Lazeron RHC. Markers for different glial cell responses in multiple sclerosis: clinical and pathological correlations. Brain. (2002) 125:1462–73. 10.1093/brain/awf165 12076997

[107] Axelsson M Malmeström C Nilsson S Haghighi S Rosengren L Lycke J. Glial fibrillary acidic protein: a potential biomarker for progression in multiple sclerosis. J Neurol. (2011) 258:882–8. 10.1007/s00415-010-5863-2 21197541

[108] Martínez MAM Olsson B Bau L Matas E Cobo Calvo Á Andreasson U. Glial and neuronal markers in cerebrospinal fluid predict progression in multiple sclerosis. Mult Scler. (2015) 21:550–61. 10.1177/1352458514549397 25732842 PMC4390605

[109] Salzer J Svenningsson A Sundström P. Neurofilament light as a prognostic marker in multiple sclerosis. Mult Scler. (2010) 16:287–92. 10.1177/1352458509359725 20086018

[110] Cantó E Barro C Zhao C Caillier SJ Michalak Z Bove R. Association between serum neurofilament light chain levels and long-term disease course among patients with multiple sclerosis followed up for 12 years. JAMA Neurol. (2019) 76:1359–66. 10.1001/jamaneurol.2019.2137 31403661 PMC6692664

[111] Pérez-Miralles F Prefasi D García-Merino A Gascón-Giménez F Medrano N Castillo-Villalba J. CSF chitinase 3-like-1 association with disability of primary progressive MS. Neurol Neuroimmunol Neuroinflamm. (2020) 7:e815. 10.1212/NXI.0000000000000815 32611760 PMC7357419

[112] Barro C Benkert P Disanto G Tsagkas C Amann M Naegelin Y. Serum neurofilament as a predictor of disease worsening and brain and spinal cord atrophy in multiple sclerosis. Brain. (2018) 141:2382–91. 10.1093/brain/awy154 29860296

[113] Disanto G Barro C Benkert P Naegelin Y Schädelin S Giardiello A. Serum Neurofilament light: a biomarker of neuronal damage in multiple sclerosis. Ann Neurol. (2017) 81:857–70. 10.1002/ana.24954 28512753 PMC5519945

[114] Martin S-J McGlasson S Hunt D Overell J. Cerebrospinal fluid neurofilament light chain in multiple sclerosis and its subtypes: a meta-analysis of case-control studies. J Neurol Neurosurg Psychiatry. (2019) 90:1059–67. 10.1136/jnnp-2018-319190 31123141 PMC6820150

[115] Petzold A de Boer JF Schippling S Vermersch P Kardon R Green A. Optical coherence tomography in multiple sclerosis: a systematic review and meta-analysis. Lancet Neurol. (2010) 9:921–32. 10.1016/S1474-4422(10)70168-X 20723847

[116] Petzold A Balcer LJ Calabresi PA Costello F Frohman TC Frohman EM. Retinal layer segmentation in multiple sclerosis: a systematic review and meta-analysis. Lancet Neurol. (2017) 16:797−812. 10.1016/S1474-4422(17)30278-8 28920886

[117] Martinez-Lapiscina EH Arnow S Wilson JA Saidha S Preiningerova JL Oberwahrenbrock T. Retinal thickness measured with optical coherence tomography and risk of disability worsening in multiple sclerosis: a cohort study. Lancet Neurol. (2016) 15:574–84. 10.1016/S1474-4422(16)00068-5 27011339

[118] Bsteh G Hegen H Teuchner B Amprosi M Berek K Ladstätter F. Peripapillary retinal nerve fibre layer as measured by optical coherence tomography is a prognostic biomarker not only for physical but also for cognitive disability progression in multiple sclerosis. Mult Scler. (2019) 25:196–203. 10.1177/1352458517740216 29095097

[119] Baldassari LE Fox RJ. Therapeutic advances and challenges in the treatment of progressive multiple sclerosis. Drugs. (2018) 78:1549–66. 10.1007/s40265-018-0984-5 30255442

[120] Correale J Gaitán MI Ysrraelit MC Fiol MP. Progressive multiple sclerosis: from pathogenic mechanisms to treatment. Brain. (2017) 140:527–46. 10.1093/brain/aww258 27794524

[121] Lassmann H. Targets of therapy in progressive MS. Mult Scler. (2017) 23:1593–9. 10.1177/1352458517729455 29041864

[122] Kappos L Bar-Or A Cree BAC Fox RJ Giovannoni G Gold R. Siponimod versus placebo in secondary progressive multiple sclerosis (EXPAND): a double-blind, randomised, phase 3 study. Lancet. (2018) 391:1263–73. 29576505 10.1016/S0140-6736(18)30475-6

[123] Castillo-Trivino T Braithwaite D Bacchetti P Waubant E. Rituximab in relapsing and progressive forms of multiple sclerosis: a systematic review. PLoS One. (2013) 8:e66308. 10.1371/journal.pone.0066308 23843952 PMC3699597

[124] Hawker K O'Connor P Freedman MS Calabresi PA Antel J Simon J. Rituximab in patients with primary progressive multiple sclerosis: results of a randomized double-blind placebo-controlled multicenter trial. Ann Neurol. (2009) 66:460–71. 10.1002/ana.21867 19847908

[125] European Medicines Agency Website. EPAR IFN beta 1b sc. Available online at: https://www.ema.europa.eu/en/documents/product-information/extavia-epar-product-information_en.pdf (accessed June 17, 2020).

[126] European Medicines Agency Website. EPAR IFN beta 1a sc. Available online at: https://www.ema.europa.eu/en/documents/product-information/rebif-epar-product-information_en.pdf (accessed June 17, 2020).

[127] European Medicines Agency Website. EPAR Mitoxantrone. Available online at: https://www.ema.europa.eu/en/documents/referral/novantrone-article-30-referral-annex-iii_en.pdf (accessed June 17, 2020).

[128] European Medicines Agency Website. EPAR Ocrelizumab. Available online at: https://www.ema.europa.eu/en/documents/product-information/ocrevus-epar-product-information_en.pdf (accessed June 17, 2020).

[129] European Medicines Agency Website. EPAR Siponimod. Available online at: https://www.ema.europa.eu/en/medicines/human/EPAR/mayzent (Accessed June 17, 2020).

[130] La Mantia L Vacchi L Rovaris M Di Pietrantonj C Ebers G Fredrikson S. Interferon β for secondary progressive multiple sclerosis: a systematic review. J Neurol Neurosurg Psychiatry. (2013) 84:420–26. 10.1136/jnnp-2012-303291 22952326

[131] Leary SM Miller DH Stevenson VL Brex PA Chard DT Thompson AJ. Interferon β-1a in primary progressive MS: an exploratory, randomized, controlled trial. Neurology. (2003) 60:44–51. 10.1212/WNL.60.1.44 12525716

[132] Montalban X Sastre-Garriga J Tintoré M Brieva L Aymerich FX Río J. A single-center, randomized, double-blind, placebo-controlled study of interferon beta-1b on primary progressive and transitional multiple sclerosis. Mult Scler. (2009) 15:1195–205. 10.1177/1352458509106937 19797261

[133] Ziemssen T Rauer S Stadelmann C Henze T Koehler J Penner IK. Evaluation of study and patient characteristics of clinical studies in primary progressive multiple sclerosis: A systematic review. PLoS ONE. (2015) 10:e0138243. 10.1371/journal.pone.0138243 26393519 PMC4578855

[134] Lublin F Miller DH Freedman MS Cree BAC Wolinsky JS Weiner H. Oral fingolimod in primary progressive multiple sclerosis (INFORMS): a phase 3, randomised, double-blind, placebo-controlled trial. Lancet. (2016) 387:1075–84. 10.1016/S0140-6736(15)01314-8 26827074

[135] Tur C Montalban X. Progressive MS trials: lessons learned. Mult Scler. (2017) 23:1583–92. 10.1177/1352458517729460 29041872

[136] Kapoor R Ho PR Campbell N Chang I Deykin A Forrestal F. Effect of natalizumab on disease progression in secondary progressive multiple sclerosis (ASCEND): a phase 3, randomised, double-blind, placebo-controlled trial with an open-label extension. Lancet Neurol. (2018) 17:405–15. 10.1016/S1474-4422(18)30069-3 29545067

[137] Montalban X Hauser SL Kappos L Arnold DL Bar-Or A Comi G. Ocrelizumab versus placebo in primary progressive multiple sclerosis. N Engl J Med. (2017) 376:209–20. 10.1056/NEJMoa1606468 28002688

[138] Wolinsky JS Arnold DL Brochet B Hartung HP Montalban X Naismith RT. Long-term follow-up from the ORATORIO trial of ocrelizumab for primary progressive multiple sclerosis: a post-hoc analysis from the ongoing open-label extension of the randomised, placebo-controlled, phase 3 trial. Lancet Neurol. (2020) 19:998–1009. 10.1016/S1474-4422(20)30342-2 33129442

[139] Rae-Grant A Day GS Marrie RA Rabinstein A Cree BAC. Practice guideline recommendations summary: disease-modifying therapies for adults with multiple sclerosis: report of the guideline development, dissemination, and implementation subcommittee of the american academy of neurology. Neurology. (2018) 90:777−88. 10.1212/WNL.0000000000005347 29686116

